# Sustainable Protein Sources: Extraction, Structural Characterization, and Functional Properties

**DOI:** 10.3390/foods15081357

**Published:** 2026-04-13

**Authors:** Armin Mirzapour-Kouhdasht, Jen-Yi Huang

**Affiliations:** 1 Department of Food Science and Technology, The Ohio State University, Columbus, OH 43210, USA; huang.6338@osu.edu; 2 Department of Food, Agricultural, and Biological Engineering, The Ohio State University, Columbus, OH 43210, USA

The worldwide demand for dietary protein has risen, while environmental problems and sustainability issues with traditional animal protein production methods have increased, accelerating research efforts to explore sustainable protein sources and alternative protein sources. The Special Issue “Sustainable Protein Sources: Extraction, Structural Characterization and Functional Properties” presents recent research developments that provide solutions to essential protein extraction methods, protein structural understanding, and food system functional performance issues (Contributions 1–8).

Sustainable protein extraction methods face a prominent developmental hurdle to overcome before they can be put into use by researchers, who are developing extraction methods to provide the highest possible yields of protein while still preserving nutritional and functional values. The Special Issue provides several papers describing novel processes to extract and process materials using some of the following methods: enzymatic-assisted extraction, mechanical disruption techniques, extrusion-based processing, and optimized alkaline solubilization. The results presented reveal that customized extraction techniques will produce optimal results, while reducing the amount of energy required for production, therefore allowing for the development of more environmentally friendly and more viable protein manufacturing processes (Contributions 1–4).

In addition to extraction, it is also critical to understand how proteins are structured so that they will perform satisfactorily once added to a food product. The studies in this Special Issue utilize advanced techniques to further characterize physicochemical properties to understand how processing, fractionating, and/or modifying proteins will alter their conformational properties and intermolecular interactions. The findings from these studies collectively indicate that changes to protein secondary and tertiary structures fundamentally impact the protein’s performance characteristics, such as solubility, emulsification capacity, foam stability, and gelation (Contributions 3, 6 and 7).

In addition to techno-functional performance, nutritional quality and biological effects must also be actively considered for sustainable protein adoption. Several articles published in this Special Issue examine digestibility, bioavailability, and bioactivity of sustainable proteins and evaluate potential safety concerns (e.g., allergenicity, cytotoxicity). Strategies (e.g., germination, enzymatic hydrolysis, conjugation, protein blending) that enhance digestibility/nutritional quality, while reducing undesirable biological effects, have been identified in these studies, reinforcing the need for evaluating nutrition/safety during the protein creation pipeline (Contributions 2, 5 and 7). Various studies have produced comparable outcomes on different alternative protein sources, highlighting the impact of the processing method used to produce these proteins on their digestibility and health benefits. Such examples of protein processing methods are ultrasound-assisted extraction, enzyme hydrolysis, and protein–phenol conjugation. Evidence supports the improved digestibility of and reduced antinutritional factors in protein from plants as a consequence of using these processing methods [1,2,3,4]. Studies performed using insect-based protein sources and pseudocereal protein sources demonstrate similar results of increased nutritional quality and functional characteristics as a result of using germination and protein blending methods [5,6,7,8]. Collectively, these findings on alternative proteins identify the necessity of conducting an integrated evaluation of the proteins’ nutritional and safety aspects in conjunction with the techno-functional characteristics of the completed product throughout the entire production process of sustainable sources of protein.

The different sources of protein from multiple areas that are investigated within the studies printed in this Special Issue demonstrate the wide-ranging scope of the present sustainability sciences of protein research program. The studies consist of plant proteins, microalgal proteins, fungus-derived proteins, and hybrid systems utilizing proteins from various sources. Many of the studies review the application of these proteins in complex food matrices, including meat alternatives or analogues, protein blends, and structured food systems, highlighting the future potential of using these sustainable protein sources as a replacement for or complement to traditional protein sources in future food products (Contributions 3, 4 and 8).

There are still significant gaps in knowledge; however, this Special Issue documents substantial progress in research on sustainable proteins. Future research will need to focus on developing processing technologies that are environmentally sustainable, scalable, and low-cost, in addition to creating validated methodologies for characterizing proteins and evaluating protein functionality. In addition, there needs to be more focus on linking macroscopic and microscopic food properties to consumer attributes. Furthermore, integrating sustainable proteins into new food technology, such as advanced formulation methods and structured food design, presents an excellent future research opportunity (Contributions 6–8).

Overall, this Special Issue has provided a thorough review of new advances in sustainable protein research, which includes extracting technologies, the structural characterization of proteins, their functional performance, and the nutritional aspects. Therefore, the consolidation of the advances presented in this Special Issue will create a more thorough understanding of how to best develop and use sustainable proteins within food systems. We hope this Special Issue will serve as an important resource and inspiration for continued cross-disciplinary research focused on the development of sustainable protein solutions that will help build a resilient and secure global food supply.

## Data Availability

Not applicable.

## References

[1] Aghababaei F., McClements D.J., Hadidi M. (2024). Ultrasound processing for enhanced digestibility of plant proteins. Food Hydrocoll..

[2] Gu J., Bk A., Wu H., Lu P., Nawaz M.A., Barrow C.J., Suleria H.A.R. (2023). Impact of processing and storage on protein digestibility and bioavailability of legumes. Food Rev. Int..

[3] Yan X., Zeng Z., McClements D.J., Gong X., Yu P., Xia J., Gong D. (2023). A review of the structure, function, and application of plant-based protein–phenolic conjugates and complexes. Compr. Rev. Food Sci. Food Saf..

[4] Liu J., Yong H., Yao X., Hu H., Yun D., Xiao L. (2019). Recent advances in phenolic–protein conjugates: Synthesis, characterization, biological activities and potential applications. RSC Adv..

[5] Jin J., Ohanenye I.C., Udenigwe C.C. (2022). Buckwheat proteins: Functionality, safety, bioactivity, and prospects as alternative plant-based proteins in the food industry. Crit. Rev. Food Sci. Nutr..

[6] Karaca A.C., Nickerson M., Caggia C., Randazzo C.L., Balange A.K., Carrillo C., Capanoglu E. (2023). Nutritional and functional properties of novel protein sources. Food Rev. Int..

[7] Queiroz L.S., Silva N.F.N., Jessen F., Mohammadifar M.A., Stephani R., de Carvalho A.F., Casanova F. (2023). Edible insect as an alternative protein source: A review on the chemistry and functionalities of proteins under different processing methods. Heliyon.

[8] Huang C., Feng W., Xiong J., Wang T., Wang W., Wang C., Yang F. (2019). Impact of drying method on the nutritional value of the edible insect protein from black soldier fly (*Hermetia illucens* L.) larvae: Amino acid composition, nutritional value evaluation, in vitro digestibility, and thermal properties. Eur. Food Res. Technol..

